# Combining impact monitoring mouthguards and blood biomarkers to monitor head impacts among Muay Thai athletes – A case study

**DOI:** 10.1016/j.jsampl.2023.100044

**Published:** 2023-11-04

**Authors:** Mikael Swarén, Joel Simrén, Hanna Huber, Henrik Zetterberg

**Affiliations:** a Swedish Unit for Metrology in Sports, Department of Sports and Health Sciences, School of Health and Welfare, Dalarna University, Falun, Sweden; b Department of Psychiatry and Neurochemistry, Institute of Neuroscience & Physiology, The Sahlgrenska Academy at the University of Gothenburg, Mölndal, Sweden; c Clinical Neurochemistry Laboratory, Sahlgrenska University Hospital, Mölndal, Sweden; d Clinical Chemistry Laboratory, Sahlgrenska University Hospital, Mölndal, Sweden; e Department of Neurodegenerative Disease, UCL Institute of Neurology, Queen Square, London, UK; f UK Dementia Research Institute at UCL, London, UK; g Hong Kong Center for Neurodegenerative Diseases, Clear Water Bay, Hong Kong, China; h Wisconsin Alzheimer's Disease Research Center, University of Wisconsin School of Medicine and Public Health, University of Wisconsin–Madison, Madison, WI, USA

**Keywords:** Sports related concussion, Sports, Neurofilament light, Martial arts

## Abstract

**Objective:**

To investigate the relationship between head impact characteristics and the levels of blood biomarkers associated with brain injury, neurofilament light (NfL) and glial fibrillary acidic protein (GFAP).

**Methods:**

Four elite amateur Muay Thai athletes were equipped with impact monitoring mouthguards, collecting linear and rotational acceleration data during a period of eight weeks. Capillary blood samples were collected after each period of sparring sessions to analyse the levels of NfL and GFAP.

**Results:**

On a group level, mean GFAP levels were negatively correlated to mean impacts per session (p ​< ​0.05). Two athletes had significant correlations between head impact characteristics and the levels of NfL and/or GFAP.

**Conclusions:**

The results indicate that NfL and GFAP might responded differently to linear and rotational accelerations and/or that the effect of different types of accelerations on brain tissue integrity is individual. The methods used could be useful to monitor brain health in different impacts sports.

## 1 Introduction

Despite sports-related concussions being common, there is a lack of objective methods for diagnosing the degree of brain injury and monitoring the recovery process to ensure that the person has recovered and is ready for physical activities again (return to play). It is known that returning to physical activity or competition too soon after a concussion prolongs the recovery time [1].

Previous studies have shown that the biomarker neurofilament light (NfL) can be used to differentiate head injury with and without neuroaxonal injury when measured in cerebrospinal fluid (CSF) [[2], [3], [4]] and in blood [5]. It has also been shown that NfL and glial fibrillary acidic protein (GFAP) are elevated in boxers who have received repeated blows to the head, even though they have rested for up to 14 days [3,4]. Longitudinal monitoring of blood biomarkers may provide help in giving athletes recommendations about when to ‘‘return-to-play’’ after sports related concussions [[6], [7], [8], [9]].

Combat sports athletes are constantly exposed to repetitive head impacts and previous studies [4,[10], [11], [12], [13]] have investigated head impact exposure and/or the consequences of repetitive impacts e.g., in boxing and MMA. However, there are still knowledge and behavior gaps among combat sports athletes and coaches, on the potential impacts of concussion [14].

To best of the authors’ knowledge, no previous study has investigated head impact exposure among Muay Thai athletes nor combining head impact data with blood biomarkers. Hence, the aim of the present study was to equipe elite amateur Muay Thai athletes with impact monitoring mouthguards, to analyse the relationship between head impact characteristics and levels of the blood biomarkers NfL and GFAP.

## 2 Method

Four Swedish elite amateur Muay Thai athletes were recruited to participate in the study (age 33 ​± ​4 years, body weight 66 ​± ​6 ​kg, women n ​= ​3, men n ​= ​1). The Swedish ethical board preapproved the study and experimental protocol (#2022-01810-01) and the study was conducted in accordance with the Declaration of Helsinki. All participants where fully informed of the nature of the study through written and verbal information before consenting to participate.

All participants were equipped with an individually fitted “boil and bite” impact monitoring mouthguards (IMM) (Prevent Biometrics, Edina, MN, USA), sampling linear and rotational acceleration and velocity data at 3200 ​Hz, with a measurement range of ±200 ​g and ±35 ​rad/s. The impact trigger threshold requires a single raw sample >5 ​g on any single axis of the accelerometer data, including dynamic head movements caused by e.g., body hits. The 5 ​g impact threshold was used to include indirect head acceleration events, as suggested by Tooby et al. [15]. The linear and rotational acceleration data from the IMMs were automatically transformed to the estimated cranial center of mass by Prevent Biometrics’ software which also estimates the impact location based on pre-defined areas.

Noviplex™ Plasma Prep Cards were used to collect blood capillary plasma samples obtain with a finger prick, from each athlete, every second week. NfL and GFAP were quantified by Single molecule array (Simoa) technology [16] using commercially available kits, and compared to peak linear accelerations (PLA), peak angular accelerations (PAA), peak linear velocities (PLV), peak angular velocities (PAV), workload per impact, accumulated workload and number of impacts for the corresponding time period.

The data collection lasted eight weeks and the athletes were instructed to perform their training and sparring sessions as normal.

All group impact data were pre-checked for normality using Shapiro–Wilk test showing that none of the group impact data conformed to normal distribution (Shapiro–Wilk test, p ​< ​0.05). Individual data were analysed using linear regression, with an omnibus analysis of variance (ANOVA) test. Comparisons between athletes regarding head impact characteristics were performed using a Kruskal–Wallis test with epsilon squared (ε^2^) for determination of effect size. A Dwass-Steel-Critchlow-Flinger test was applied for pairwise comparisons if there was a global significance for the Kruskal–Wallis test. All statistical analyses were performed using *jamovi* [17].

## 3 Results

In total, 1223 impacts were registered by the IMGs during 44 sessions. There was an overall difference between athletes for PLA (χ^2^ (3) ​= ​28.83, p ​< ​0.001, ε^2^ ​= ​0.02359), and for PLV (χ^2^ (3) ​= ​33.07, p ​< ​0.001, ε^2^ ​= ​0.02706). Athlete B had significantly higher PLA compared to Athlete A, Athlete C and Athlete D (p ​< ​0.001, p ​< ​0.001 and p ​= ​0.039, respectively). Athlete A had significantly higher PLV compared to Athlete C and Athlete D (p ​< ​0.001 and p ​= ​0.035, respectively). Athlete B had significantly higher PLV compared to Athlete C and Athlete D (p ​< ​0.001 and p ​= ​0.031, respectively), and Athlete D had significantly higher PLV compared to Athlete C (p ​= ​0.023). See Table 1 for complete descriptives of head impact characteristics and Table 2 for individual results.

**Table 1:** Descriptives of all collected impacts.

	Number	PLA (g)		PAA (rad/s^2^)		PLV (m/s)		PAV (rad/sec)		Workload (J)	
	*N*	*Median*	*IQR*	*Median*	*IQR*	*Median*	*IQR*	*Median*	*IQR*	*Median*	*IQR*
Bottom Rear	113	9.9	4.8	873	619	0.5	0.3	5.7	5.2	1.5	2.4
Front Low	207	9.7	4.8	753	545	0.6	0.4	5.4	3.9	1.7	2.1
Front High	457	12.5	7.7	869	636	0.8	0.5	6.6	4.9	2.7	3.6
Bottom Front	64	9.9	4.2	830	462	0.6	0.3	5.5	4.7	1.5	2.7
Left Low	99	10.8	5.5	992	553	0.7	0.5	5.9	5.4	1.7	2.3
Right Low	57	9.2	5.2	916	567	0.5	0.3	6.4	3.2	1.3	1.9
Rear Low	18	12.4	6.0	1167	429	0.7	0.3	6.2	5.0	1.6	2.4
Left High	55	13.5	6.3	1063	690	1.0	0.6	7.9	5.4	3.5	3.7
Top Front	95	12.0	7.3	934	849	0.7	0.4	6.9	4.3	3.3	3.9
Right High	39	11.6	7.6	930	636	0.9	0.5	7.7	4.2	3.3	3.1
Top Rear	7	13.0	6.1	1181	483	0.9	0.9	7.9	6.1	2.9	4.4
Rear High	12	9.2	6.1	961	339	0.6	0.2	6.1	4.8	1.4	1.8
*Total*	*1223*	*11.3*	6.6	*893*	617	*0.7*	*0.5*	*6.3*	*5.0*	*2.2*	*3.2*
*Maximum*	*-*	*55.0*	–	*6545*	–	*2.3*	*-*	*35.2*	*-*	*48.4*	*-*

PLA ​= ​peak linear acceleration, PAA ​= ​peak angular acceleration, PLV ​= ​peak linear velocity, PAV ​= ​peak angular velocity.

**Table 2:** Individual results for blood biomarkers and head impact characteristics.

	Athlete A	Athlete B	Athlete C	Athlete D
Mean NfL	0.33 ​± ​0.19	0.44 ​± ​0.12	0.24 ​± ​0.06	0.40 ​± ​0.11
Mean GFAP	2.14 ​± ​0.65	3.34 ​± ​2.10	3.42 ​± ​0.97	3.95 ​± ​0.81
Median PLA (g)	10.8 [6.0]	12.0 [8.0]	9.9 [4.8]	10.8 [5.6]
Max PLA (g)	37.0	65.0	25.5	40.5
Median PAA (rad/s^2^)	872 [527]	897 [701]	895 [636]	904 [619]
Max PAA (rad/s^2^)	3624	6545	2267	3419
Median PLV (m/s)	0.7 [0.5]	0.7 [0.5]	0.5 [0.4]	0.6 [0.3]
Max PLV (m/s)	2.0	2.3	1.4	2.2
Median PAV (rad/s)	6.1 [4.5]	6.6 [5.2]	7.1 [5.2]	6.1 [4.25]
Max PAV (rad/s)	26.3	35.2	18.3	22.7
Median workload per impact (J)	2.1 [3.0]	2.2 [3.2]	1.8 [2.9]	2.5 [3.45]
Max workload per impact (J)	30.4	48.4	13.9	15.9
Total number of impacts	459	538	75	151

Values are presented as n, mean ​± ​SD or median [IQR]. NfL - neurofilament light; GFAP - glial fibrillary acidic protein; PLA - peak linear acceleration; PAA - peak angular acceleration; PLV - peak linear velocity; PAV - peak angular velocity.

None of the recorded impacts resulted in a diagnosed concussion.

**Fig. 1:**
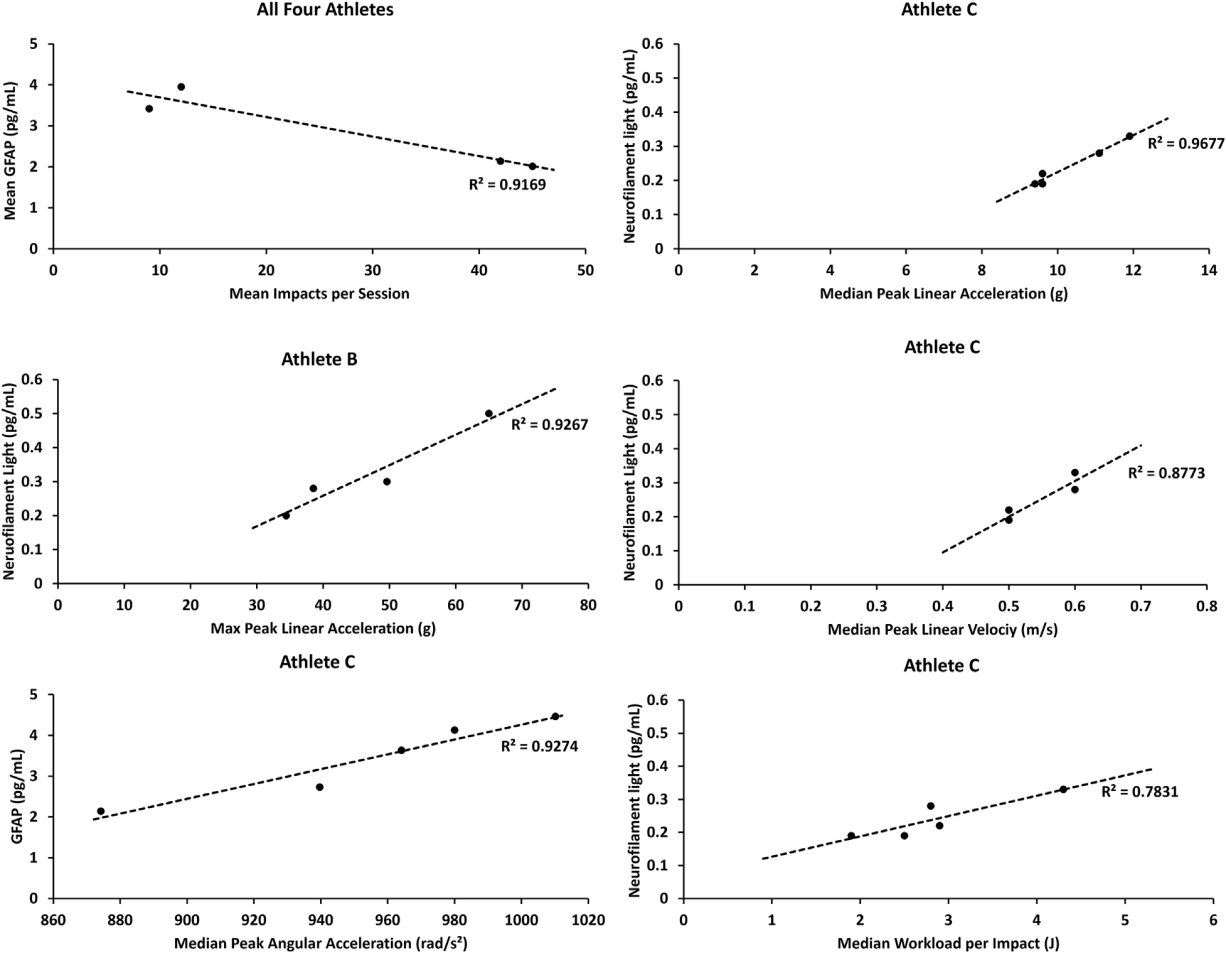
Scatterplots with regression lines for the six cases with significant correlations. GFAP ​= ​glial fibrillary acidic protein.

On a group level, mean GFAP levels were negatively associated with the mean number of head impacts per session (R^2^ ​= ​0.92, p ​< ​0.05). On an individual level, two athletes had significant correlations between head impact characteristics and the levels of NfL and/or GFAP, see Fig. 1. Athlete B showed significant correlation between NfL levels and max PLA (R^2^ ​= ​0.93, p ​< ​0.05). For Athlete C, NfL levels were significantly correlated to median PLA (R^2^ ​= ​0.97, p ​< ​0.01), median PLV (R^2^ ​= ​0.88, p ​< ​0.05) and median Workload per impact (R^2^ ​= ​0.78, p ​< ​0.05). GFAP levels for Athlete C were significantly correlated with max PAA (R^2^ ​= ​0.93, p ​< ​0.01).

## 4 Discussion

The plasma NfL and GFAP levels varied between each athlete and were affected differently by the different impact characteristics. Both Athlete B and C had significant correlations between linear acceleration values and NfL levels. Athlete C showed a relationship between angular acceleration values and GFAP. Interestingly, GFAP was negatively associated with the mean number of head impacts per sparring session. Due to the few participants, no clear explanation can be made. However, the two athletes with highest mean GFAP levels, had significantly higher PLV, compared to the other two athletes, with the lower GFAP levels. Although only four athletes and relatively few data points were included in the present study, results indicate that NfL and GFAP might respond differently to linear and rotational accelerations and/or that the effects of different types of accelerations are individual. Future research should combine IMMs and blood biomarkers on larger populations of combat sports athletes, to better understand if NfL and GFAP are affected differently by linear and rotational accelerations and/or if the response is individual.

The present PLA and PAA results are lower compared to the findings for MMA athletes by Jansen et al. [10] and for collegiate boxers by Doan et al. [11]. This is most likely due to the lower threshold in the current study, >5 ​g compared to 15 ​g and 9.6 ​g used by Jansen et al. and Doan et al., respectively [10,11]. King et al. [18] reported 10 ​g to be the most commonly used threshold and recommended future studies to use a 10 ​g threshold until more in-filed data are available. However, in a more recent study by Tooby et al. [15], both 5 ​g and 10 ​g thresholds were investigated, using rugby players in the field. They found that a 5 ​g threshold is better for field tests as more indirect head acceleration events are included in the data set. As hits and kicks to the body are essential in Muay Thai, a 5 ​g threshold was used to include indirect head acceleration events, and not just direct head impacts.

It is important to objectively be able to measure the violence to the head and the size of the brain injury to make a correct and objective assessment of the size of the brain injury and knowing when an athlete can resume training/competition (a.k.a. return to play). The IMMs provide accurate impact data for everyday monitoring and can be used without video assessment of false positives/negative impacts [19,20]. Also, as suggested by previous research [5,8,9], longitudinal monitoring of blood biomarkers, such as NfL and GFAP, may provide athletes with recommendations about when to return to play. The combination of IMM and Noviplex™ Plasma Prep Cards shows promising results for monitoring the brain health among Muay Thai athletes and should be evaluated in other impact sports, e.g., ice hockey, rugby, boxing and American football, as there is no need of direct access to blood centrifuges and freezer for the handling of the blood samples.

Overall, the results from the current case study indicates that NfL and GFAP might respond differently to linear and rotational accelerations and/or that the effects of different types of accelerations are individual. The combination of IMM and Noviplex™ Plasma Prep Cards to monitor head impacts and brain damage should be evaluated in other impact sports.

## Ethical statement

This research is not part of a larger study, and this manuscript represents results of original work that have not been published elsewhere. This manuscript has not and will not be submitted for publication elsewhere until a decision is made regarding its acceptability for publication in the Journal of Science and Medicine in Sport Plus. If accepted for publication, it will not be published elsewhere.

The Swedish ethical board preapproved the study and experimental protocol (#2022-01810-01) and the study was conducted in accordance with the Declaration of Helsinki. All participants where fully informed of the nature of the study through written and verbal information before consenting to participate. All authors acknowledge ethical responsibility for the content of the manuscript and will accept the consequences of any ethical violation.

## Funding details

This work was supported by the Swedish Research Council for Sport Science under Grant CIF 2021/9 P2022-0077. Henrik Zetterberg is a Wallenberg Scholar supported by grants from the Swedish Research Council (#2022-01018 and #2019-02397), the European Union's Horizon Europe research and innovation programme under grant agreement No 101053962, Swedish State Support for Clinical Research (#ALFGBG-71320), the Alzheimer Drug Discovery Foundation (ADDF), USA (#201809-2016862), the AD Strategic Fund and the Alzheimer's Association (#ADSF-21-831376-C, #ADSF-21-831381-C, and #ADSF-21-831377-C), the Bluefield Project, the Olav Thon Foundation, the Erling-Persson Family Foundation, Stiftelsen för Gamla Tjänarinnor, Hjärnfonden, Sweden (#FO2022-0270), the European Union's Horizon 2020 research and innovation programme under the Marie Skłodowska-Curie grant agreement No ne, the European Union Joint Programme – 10.13039/100013278Neurodegenerative Disease Research (JPND2021-00694), the National Institute for Health and Care Research University College London Hospitals Biomedical Research Centre, and the 10.13039/501100017510UK Dementia Research Institute at UCL (UKDRI-1003).

## Declaration of competing interest

Henrik Zetterberg has served at scientific advisory boards and/or as a consultant for AbbVie, Acumen, Alector, Alzinova, ALZPath, Annexon, Apellis, Artery Therapeutics, AZTherapies, CogRx, Denali, Eisai, Nervgen, Novo Nordisk, Optoceutics, Passage Bio, Pinteon Therapeutics, Prothena, Red Abbey Labs, reMYND, Roche, Samumed, Siemens Healthineers, Triplet Therapeutics, and Wave, has given lectures in symposia sponsored by Cellectricon, Fujirebio, Alzecure, Biogen, and Roche, and is a co-founder of Brain Biomarker Solutions in Gothenburg AB (BBS), which is a part of the GU Ventures Incubator Program (outside submitted work). The other authors have no conflict of interest.
